# Sustained Hemodialysis Through Thoracic Collateral Veins After Exhaustion of Conventional Vascular Access: A Case Report from a Sub-Saharan African Country

**DOI:** 10.7759/cureus.108692

**Published:** 2026-05-11

**Authors:** Cecília Agostinho, Liudmila Menezes, Cláudio Mbala, Patrick Afonso

**Affiliations:** 1 Nephrology, Clínica Multiperfil, Luanda, AGO; 2 Cardiology, Clínica Multiperfil, Luanda, AGO

**Keywords:** angiotomography, arteriovenous fistula, chronic kidney disease, hemodialysis, thoracic collateral circulation, vascular access insufficiency

## Abstract

We present the case of a 59-year-old man, on hemodialysis for 16 years, diagnosed with central venous stenosis confirmed by angiotomography and associated failure of conventional vascular access. Five years ago, the puncture of collateral veins in the chest wall was initiated as an alternative venous access for dialysis treatment. Since then, the patient has maintained satisfactory dialysis adequacy (spKt/V ≥ 1.2), demonstrating that unconventional vascular access can ensure therapeutic continuity, clinical stability, and effective long-term dialysis management.

## Introduction

Functional vascular access is necessary for all extracorporeal therapies and is essential for patients undergoing chronic hemodialysis [1]. Patients undergoing dialysis treatment may experience access-related complications over time, including venous stenosis, making treatment difficult or nearly impossible due to loss of vascular access [2-4]. In most patients, venous stenosis results from prior insertion of central venous catheters (CVCs) and presents with limb or facial edema; it can occur in up to 40% of hemodialysis patients [2,4].

Chronic kidney disease (CKD) is currently a major public health problem in Sub-Saharan Africa, with an estimated prevalence of 18% and an impact on more than 170 million affected individuals in the region [5].

In recent years, Angola has recorded a significant increase in the number of patients with CKD undergoing hemodialysis, reaching approximately 4,000 in March 2025. This phenomenon has been accompanied by a positive evolution in survival [6]. For these patients, functional vascular access is essential, although the conventional sites available for vascular access are limited, and vascular bed depletion constitutes a serious and environmentally fatal complication [7].

Furthermore, in Sub-Saharan African countries, limited resources and restricted access to alternative therapies, such as peritoneal dialysis and kidney transplantation, exacerbate the impact of impaired vascular access, since a proportion of patients are unable to consistently access hemodialysis [5].

In Angola, these challenges are particularly evident, reflecting limitations in the availability of alternative techniques, specialized human resources, and infrastructure for advanced vascular access management, such as trans-lumbar or transhepatic accesses [8], making vascular bed depletion a critical challenge for the continuity of hemodialysis, associated with increased morbidity and risk of mortality.

In this context, it becomes essential to consider alternative vascular access strategies. This case report describes a situation of vascular access failure, highlighting the clinical challenge, as well as the clinical relevance of less conventional approaches. Reports of similar strategies remain limited, particularly within Sub-Saharan Africa.

## Case presentation

A 59-year-old black male patient, a former smoker, with a history of long-standing hypertension and CKD of undetermined etiology, has been on hemodialysis for 16 years. He initially used a temporary CVC in the right femoral vein, which was removed due to infection after four months and replaced with a CVC in the left femoral vein.

Due to dysfunction of the femoral accesses, a long-term catheter was subsequently implanted in the right internal jugular vein. During this period, a left radiocephalic arteriovenous fistula (AVF) was created, which evolved with primary failure, and a left brachiocephalic AVF, which evolved with thrombosis after several years of adequate functioning.

Given the need to maintain dialysis therapy, new femoral and jugular CVCs were attempted alternately, but presented insufficient flows or the impossibility of guidewire progression. The evaluation for AVF creation in the right upper limb demonstrated the absence of favorable anatomical and hemodynamic criteria for functional access. Given the critical situation, transition to peritoneal dialysis was considered, but the patient refused.

**Figure 1:**
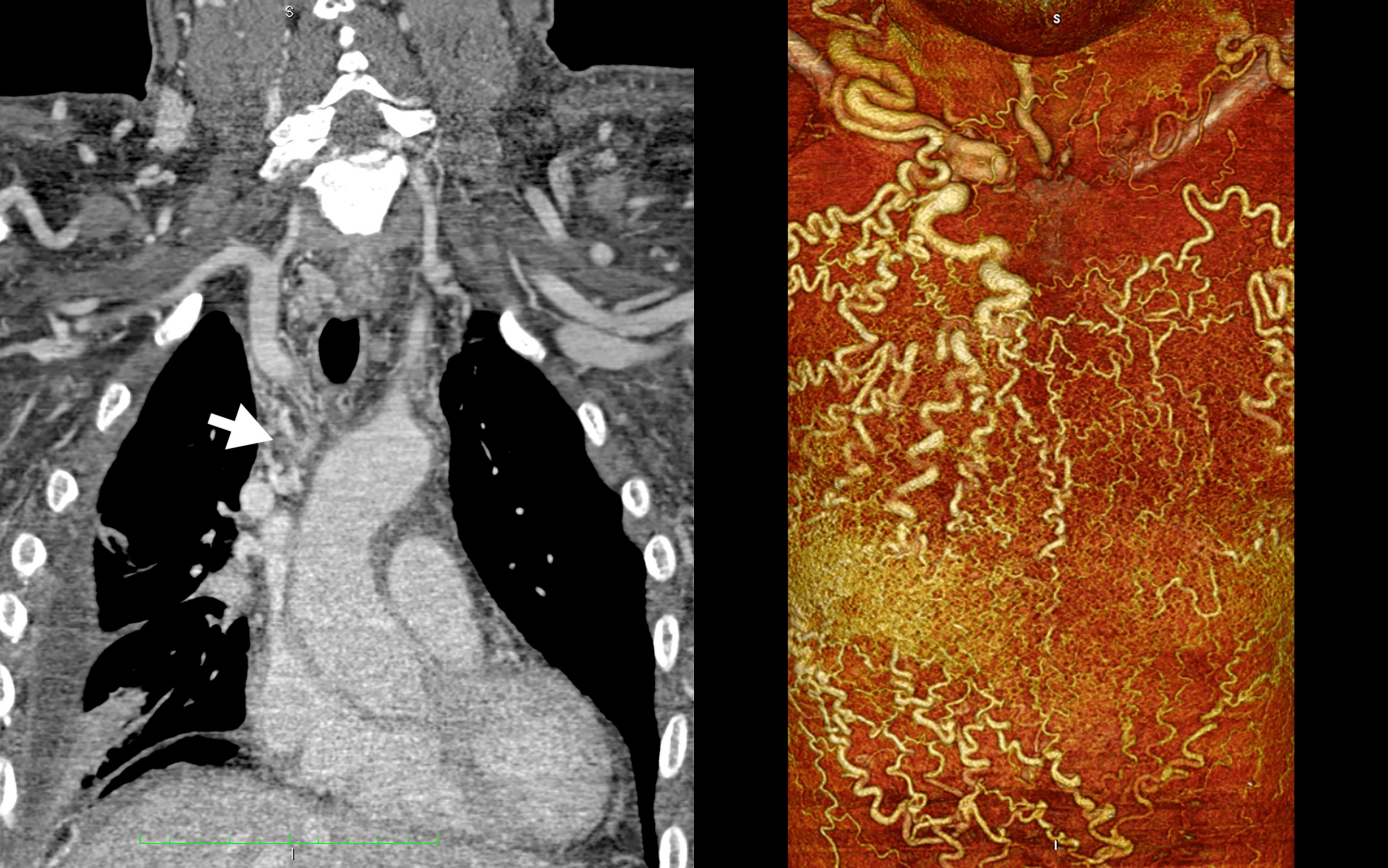
Angiography showing the site of venous occlusion in the superior vena cava (arrow, left image). Three-dimensional reconstruction demonstrating thoracic and cervical collateral circulation (right image).

Subsequently, the appearance of prominent thoracic collateral veins and facial edema was observed. An angiotomography (angio CT) confirmed central venous stenosis (Figure 1), without the possibility of endovascular intervention due to technical limitations.

**Figure 2:**
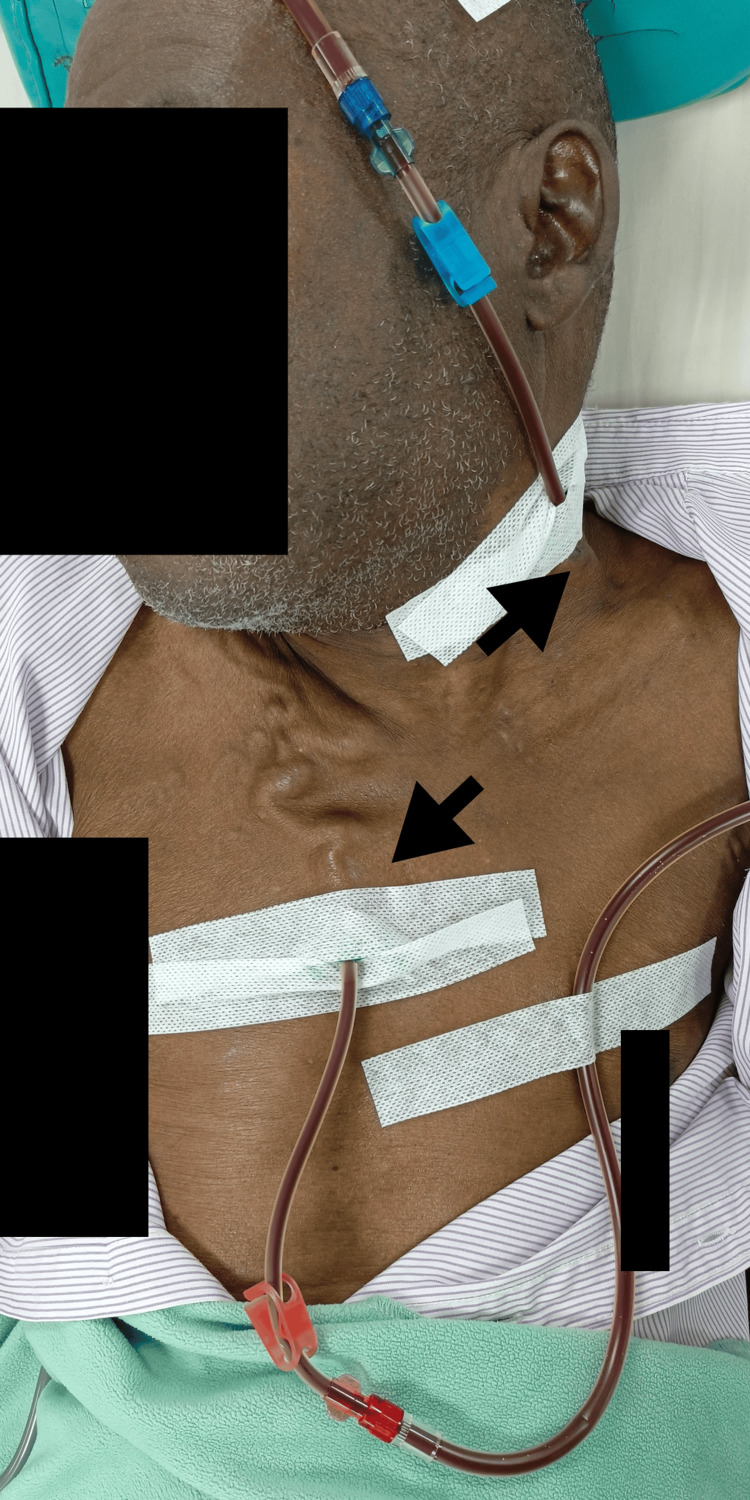
Current puncture sites (arrows) of the patient.

**Figure 3:**
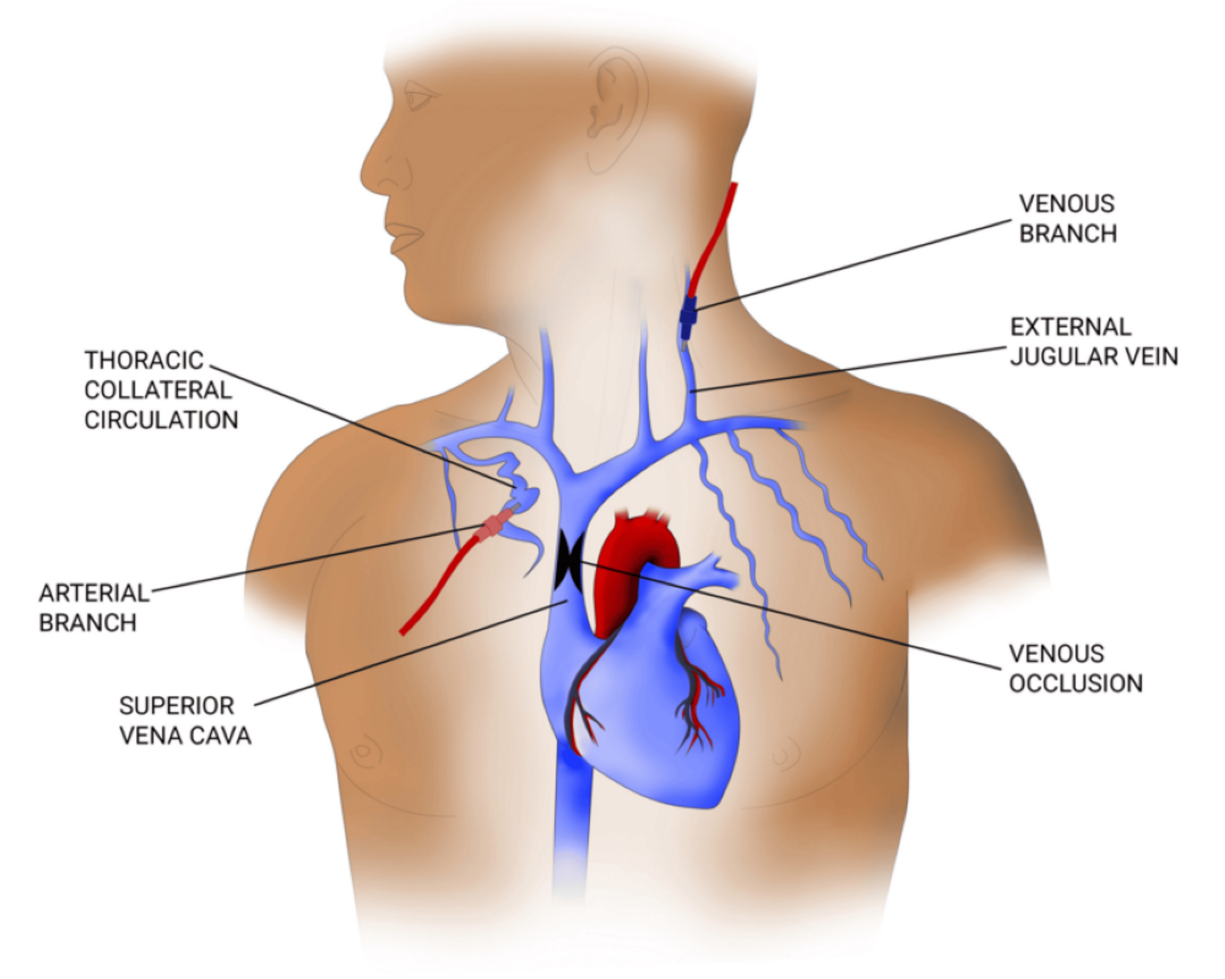
Schematic representation of superior vena cava occlusion, thoracic collateral venous circulation and hemodialysis cannulation sites. Created by Patrick L. Afonso using Adobe Photoshop 2020, Luanda, Angola.

Given the impossibility of establishing conventional access, the team opted for an alternative strategy, namely, puncture of the collateral circulation, using a similar technique and aseptic procedure as for AVF access, with 16G and 20 mm dialysis needles. The arterial branch was established in a collateral vein of the thoracic wall, and the venous branch in the external jugular vein (Figures 2-3). This approach allowed maintaining dialysis treatment with a blood flow of 250 mL/minute, with arterial and venous pressures remaining within appropriate limits, indicating adequate vascular access function and effective dialysis delivery, sufficient to achieve single pool Kt/V (spKt/V) measured by online clearance monitor ≥ 1.2, ranging from 1.23 to 1.79 (Table 1).

**Table 1:** Dialysis prescription and adequacy parameters at the most recent evaluation. URR, urea reduction ratio

Parameters	Value
Session duration	3.5 hours
Frequency	3 sessions/week
Blood flow	250 mL/minute
Dialysate flow	400 mL/minute
Dialyzer	Diacap 13
Dialysate K+	2.0 mEq/L
Dialysate Ca++	1.25 mEq/L
Dialysate Na+	138 mEq/L
Heparin	250 UI/hour
Pre-dialysis urea	82.01 mg/dL
Post-dialysis urea	16.22 mg/dL
URR	80.2%
spKt/V	1.55
Serum calcium	8.34 mg/dL
Serum phosphorus	3.81 mg/dL
Serum potassium	5.55 mg/dL

The patient remains on hemodialysis using this technique, maintaining clinical and hemodynamic stability, with no episodes of intradialytic hypotension and adequate volume status, without evidence of fluid overload or depletion. This therapeutic approach has been sustained for five years through the use of unconventional vascular access, without technical, thrombotic, or infectious complications related to the access.

## Discussion

Vascular access is crucial for the effectiveness of hemodialysis. Complications such as infection, thrombotic events, and central stenosis compromise the patency of the access, and consequently the dialysis effectiveness, and the patient's survival [1,9].

In the case presented, therapy time, infectious episodes, access changes, and endothelial trauma are plausible factors for the development of central stenosis [2,9,10]. The emergence of collateral circulation led to the unconventional initiative of using it for hemodialysis. In the literature, case reports of unconventional access use can be found in other contexts, such as Brazil, Poland, and Egypt [7,8,11,12]. In two of these reports, patients utilized abdominal collateral veins; however, one patient died shortly after initiation of therapy due to causes not related to the vascular access, while the other report did not provide follow-up data [7,11].

In this patient, the puncture and use of thoracic collateral veins as an alternative vascular access represented a novel approach in the local context, which is particularly relevant in an environment with limited technical resources, where alternative approaches are not usually available [13]. In this context, published experience with this type of unconventional vascular access in Sub-Saharan Africa appears limited, supporting the unique and promising nature of this approach.

The patient maintains satisfactory dialysis parameters, demonstrating the functional viability of the approach adopted.

## Conclusions

The case presented demonstrates that, in situations of exhaustion of the conventional vascular bed, the use of unconventional access can constitute a viable alternative to guarantee the continuity of hemodialysis. Puncture of thoracic collaterals proved to be functional and ensured therapy with satisfactory adequacy.

Additionally, the case reinforces the importance of planning and preserving the vascular bed from the beginning of dialysis therapy, as well as considering and promoting investment in alternative and individualized approaches in selected cases, particularly in resource-limited settings.
